# Metabolomic basis of laboratory evolution of butanol tolerance in photosynthetic *Synechocystis* sp. PCC 6803

**DOI:** 10.1186/s12934-014-0151-y

**Published:** 2014-11-01

**Authors:** Yaxing Wang, Mengliang Shi, Xiangfeng Niu, Xiaoqing Zhang, Lianju Gao, Lei Chen, Jiangxin Wang, Weiwen Zhang

**Affiliations:** Laboratory of Synthetic Microbiology, School of Chemical Engineering & Technology, Tianjin University, Tianjin, 300072 P.R. China; Key Laboratory of Systems Bioengineering, Ministry of Education of China, Tianjin, 300072 P.R. China; Collaborative Innovation Center of Chemical Science and Engineering, Tianjin, P.R. China

**Keywords:** Butanol, Tolerance, Evolution, Metabolomics, *Synechocystis*

## Abstract

**Background:**

Recent efforts demonstrated the potential application of cyanobacteria as a “microbial cell factory” to produce butanol directly from CO_2_. However, cyanobacteria have very low tolerance to the toxic butanol, which limits the economic viability of this renewable system.

**Results:**

Through a long-term experimental evolution process, we achieved a 150% increase of the butanol tolerance in a model cyanobacterium *Synechocystis* sp. PCC 6803 after a continuous 94 passages for 395 days in BG11 media amended with gradually increased butanol concentration from 0.2% to 0.5% (*v*/*v*). To decipher the molecular mechanism responsible for the tolerance increase, we employed an integrated GC-MS and LC-MS approach to determine metabolomic profiles of the butanol-tolerant *Synechocystis* strains isolated from several stages of the evolution, and then applied PCA and WGCNA network analyses to identify the key metabolites and metabolic modules related to the increased tolerance. The results showed that unstable metabolites of 3-phosphoglyceric acid (3PG), *D*-fructose 6-phosphate (F6P), *D*-glucose 6-phosphate (G6P), NADPH, phosphoenolpyruvic acid (PEP), *D*-ribose 5-phosphate (R5P), and stable metabolites of glycerol, *L*-serine and stearic acid were differentially regulated during the evolution process, which could be related to tolerance increase to butanol in *Synechocystis*.

**Conclusions:**

The study provided the first time-series description of the metabolomic changes related to the gradual increase of butanol tolerance, and revealed a metabolomic basis important for rational tolerance engineering in *Synechocystis*.

**Electronic supplementary material:**

The online version of this article (doi:10.1186/s12934-014-0151-y) contains supplementary material, which is available to authorized users.

## Background

Due to its superior chemical properties in terms of energy content, volatility, corrosiveness and its compatibility with the existing fuel storage and distribution infrastructure, butanol has been proposed as a next-generation transportation biofuel to substitute or supplement gasoline [1,2]. In addition to continuous efforts to improve butanol production in various native butanol-producing microbes [3-5], pioneer attempts have been made in recent years to employ photosynthetic cyanobacteria as a carbon-neutral ‘microbial factories’ to produce biofuel butanol directly from CO_2_ and solar energy [6-9]. For example, Lan and Liao (2011) constructed a modified CoA-dependent 1-butanol production pathway in cyanobacterial *Synechococcus elongatus* PCC7942 and achieved 14.5 mg/L 1-butanol production in seven days directly from CO_2_ and light [8]. Further efforts by artificially engineering ATP consumption through a pathway modification can drive the thermodynamically unfavorable condensation of two molecules of acetyl-CoA to acetoacetyl-CoA forward and enable the direct photosynthetic production of 1-butanol from cyanobacteria *S. elongatus* PCC 7942. In addition, by replace the bifunctional aldehyde/alcohol dehydrogenase (AdhE2) with separate butyraldehyde dehydrogenase (Bldh) and NADPH-dependent alcohol dehydrogenase (YqhD) further increased 1-butanol production by 4-fold. Finally, the recombinant cyanobacteria strain achieved a production level of 29.9 mg/L 1-butanol [9].

Compared with the native producers, such as *Clostridium* [6], current butanol productivity from these renewable cyanobacterial systems is still very low [10]. Although the low productivity can be attributed to many biological factors (*i.e.*, gene expression, enzymatic activity and stability, and product exporting), low tolerance to butanol toxicity has been considered as one of the major hurdles for further improving productivity of the cyanobacterial production systems [10-12]. For example, the tolerance level of a model cyanobacterial *Synechocystis* sp. PCC 6803 (hereafter *Synechocystis*) to butanol was found to be at least 10 times lower than other microbes ever being investigated, including *Escherichia coli, Zymomonas mobilis*, *C. acetobutylicum* and yeast [13]; meanwhile, very little is known about the mechanism related to biofuel tolerance in cyanobacteria [9,10]. To address the issue, several “omics”-based studies were recently conducted to determine the transcriptional-, protein- and metabolite-level changes upon butanol stress in *Synechocystis* [13-15]. The results showed that *Synechocystis* cells tend to employ a combination of multiple cellular changes to achieve full protection against butanol toxicity [13-15], although genetic manipulation of selected butanol-responsive targets can also be used to improve butanol tolerance for some degree [15]. Considering most of current genetic manipulations involve only a limited number of genes/proteins, alternative methodologies that allow multigenic and large-scale metabolic changes need to be evaluated.

Recently, laboratory-based adaptive evolution has been proposed as a valuable mean to enrich favorable genetic changes and achieve better biofuels tolerance in various microbes [16-19]. Briefly, adaptive evolution subjects microbes to a serial or continuous cultivation for many generations to which it is not optimally adapted to select more fit genetic variants [16]. Using a long-term adaptation strategy on inhibitors and elevated temperature, Wallace-Salinas and Gorwa-Grauslund (2013) obtained a stable *Saccharomyces cerevisiae* isolate (ISO12) capable of growing and fermenting the liquid fraction of non-detoxified spruce hydrolysate at 39°C with an ethanol yield of 0.38 g ethanol per gram of hexoses [17]. Using a 180-day adaptive evolution process, Minty et al. [18] obtained several *E. coli* strains with improved isobutanol tolerance. Consistent with the complex, multigenic nature of isobutanol tolerance, further genome resequencing coupled with gene-expression analysis of the isobutanol-tolerant mutants revealed adaptations in a diversity of cellular processes; in addition, many adaptations appear to involve epistasis between different mutations, implying a rugged fitness landscape for isobutanol tolerance [18]. In a similar effort to address ethanol tolerance, Goodarzi et al. [19] used fitness profiling to measure the consequences of single-locus perturbations in the context of ethanol exposure, and a module-level computational analysis to reveal the organization of the contributing loci into cellular processes and regulatory pathways (*i.e.*, osmoregulation and cell-wall biogenesis) whose modifications significantly affect ethanol tolerance. Interestingly, the study found that a dominant component of adaptation involves metabolic rewiring that boosts intracellular ethanol degradation and assimilation [19]. Together, these studies demonstrated that experimental evolution approaches followed by various “omics” analysis could be a very efficient way to achieve tolerance to various biofuels, and to elucidate genetic/metabolic bases of its adaptation to biofuel stress.

In addition to genomics- and transcriptomics-based analyses that have been applied to the evolved strains [18,19], to fully elucidate the complex molecular mechanism associated with biofuel tolerance, it is necessary to include functional characterization and accurate quantification of all levels of gene products, mRNA, proteins and metabolites [20]. In particular, metabolomics, as a method to define the small-molecule diversity and to display differences in small molecule abundance in cells, is a very useful tool since cellular metabolites are ultimate functional entities within cells and their intracellular levels vary as a direct consequence of biofuel response [20,21]. In this study, we subjected *Synechocystis* to an adaptive evolution to a gradually elevated butanol stress for 395 days, and then applied an integrated Gas chromatography–mass spectrometry (GC-MS) based- and Liquid Chromatography-Mass Spectrometry (LC-MS) based-metabolomics to determine the time-series metabolomic changes of *Synechocystis*. The integrated analysis of LC-MS and GC-MS metabolomics allowed better coverage of both unstable and stable intercellular metabolites, and was applied to physiological study of *Synechocystis* for the first time. In addition, the Weighted Correlation Network Analysis (WGCNA) approach was applied to the metabolomic data to reveal active metabolic modules associated with the gradual tolerance increase against butanol. The results provided new insights into the metabolomic basis for butanol tolerance improvement in *Synechocystis*, and constituted valuable knowledge for the rational tolerance engineering in the future.

## Results and discussion

### Experimental evolution of butanol tolerance in *Synechocystis*

**Figure 1:**
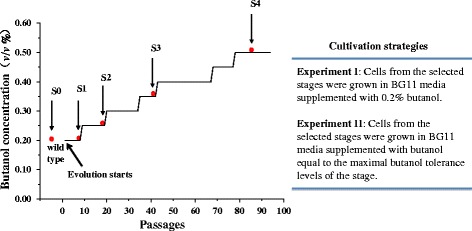
**Experimental evolution of butanol tolerance in** ***Synechocystis*** **.** The two experiment designs to cultivate samples were presented in the inserted table.

*Synechocystis* wild type was evolved by serial passaging for 94 passages in BG11 medium supplemented with butanol, as a selective pressure to enrich population with butanol tolerance. The starting butanol concentration for the wild type was set as 0.2% (*v*/*v*) as our early study showed that the strain was able to grow without significant growth deficiency at this butanol concentration level [13]. Under the typically experimental condition we established, *Synechocystis* wild type strain can reach the middle exponential phase (OD_730_ of 0.5) within 72 h in the BG11 medium without butanol stress; however, once the butanol was added, cell growth rate was decreased and it could take longer time (*i.e.*, 72–120 h) for the cells to reach OD_730_ of 0.5. In the experimental evolution process, we established a simple rule that we kept passaging the butanol-spiked cultures under one butanol concentration until their growth rates were recovered to a similar growth rate as no butanol control (*i.e.*, can reach OD_730_ of 0.5 within 72 h), and then we increased the butanol concentration by additional 0.05% (*v*/*v*). The experimental evolution proceeded for 94 passages or 395 days under butanol selective pressure, corresponding to approximately 700 generations, assuming an average ~7.5 generations per passage based on previous estimations on *Synechocystis* growth rate [22,23]. Eventually, *Synechocystis* with initial tolerate level of 0.2% (*v*/*v*) butanol was evolved through six stages of butanol adaptation (*i.e.*, 0.2, 0.25, 0.3, 0.35, 0.4, 0.45 and 0.5% of butanol) and reached an enhanced butanol tolerance level that the evolved cells have the similar growth rate under 0.5% (*v*/*v*) as that of the cells without butanol, which represents a 150% increase of butanol tolerance from the original 0.2% (*v*/*v*). Cells from several stages across the whole evolution time course were selected for further cultivation and metabolomic analyses as described in Figure 1.

### LC-MS metabolomic analysis

LC–MS based metabolomics has been increasingly applied to microbial metabolism recently [20,24], due to its advantages toward chemically unstable metabolites, such as the redox active nucleotides (NADPH, NADH) and the hydrolytically unstable nucleotides (ATP, GTP, cAMP, PEP) that are crucial for all major metabolic pathways [25-27]. More recently, LC-MS metabolomic analysis was also applied to characterize changes in the cyanobacterial primary metabolism under diverse environmental conditions or in defined mutants. The resulting identification of metabolites and their steady state concentrations has provided a better understanding of cyanobacterial metabolism [28]. In combination with other “omics” analysis, LC-MS metabolomics is expected to strengthen the base for the biotechnological application of cyanobacteria [20,28]. For example, Bennette et al. [27] developed a method of isolation and tandem LC–MS/MS quantification of a targeted subset of internal metabolites from the model cyanobacterium *Synechococcus* sp. PCC 7002. After optimization of a sampling protocol and mass spectral detection channels, screening, and optimization of chromatography, the method allowed successful monition of the intracellular levels of 25 metabolites, including intermediates in central carbon metabolism together with those involved in the cellular energy charge and redox poise [27]. We adopted the reversed-phase ion paring (RIP) method with minor modifications, including using a slower flow rate of 0.2 mL/min instead of 0.3 mL/min for *Synechococcus* [27] and individually optimizing fragmentor voltage (FV) and collision voltage (CV) for each standard metabolite using Agilent Optimizer software, and eventually established reproducible analyses for 24 selected standard metabolites, most of which are unstable metabolites in the key metabolic pathways of *Synechocystis* (Additional file 1: Table S1). Using them as references, we achieved a semi-quantitative characterization of all 24 metabolites from all butanol-evolved *Synechocystis* cell samples. The MS and MS/MS experimental parameters were optimized with the mix standard solution. The concentration of each standard metabolite used for analysis is 50 μM.

**Figure 2:**
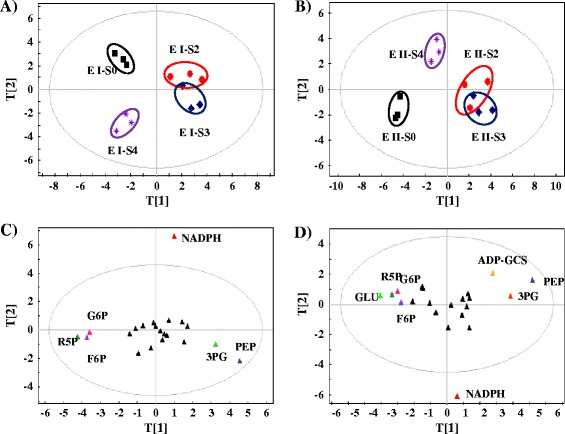
**PCA analysis of LC-MS metabolomic profiles. A)** Plot of experiment I (S0, S2, S3 and S4 cells from day 0, 72, 205 and 350 of the evolution course, corresponding to their maximal butanol tolerance of 0.2, 0.25, 0.35 and 0.5%, respectively) grown in media supplemented with 0.2% (*v*/*v*) butanol; **B)** Plot of experiment II (cells from day 0, 72, 205 and 350 of the evolution course, corresponding to their maximal butanol tolerance of 0.2, 0.25, 0.35 and 0.5%, respectively) grown in media supplemented with butanol equal to their maximal butanol tolerance levels, respectively; **C)** Loading plot of the experiment I; **D)** Loading plot of the experiment II.

With the optimized LC-MS protocol, we determined the metabolomic profiles of selected cells following the evolution time course of their tolerance increase. To fully uncover the metabolomic basis of tolerance evolution, the cells from four evolution stages were selected (S0, S2, S3, and S4 cells from day 0, 72, 205 and 350 of the evolution course, corresponding to the wild type strain and the evolved strains with the maximal butanol tolerance of 0.2, 0.25, 0.35 and 0.5%, respectively) (Figure 1), and then were re-grown and analyzed through two experimental strategies: *i*) Experiment I: S0, S2, S3, and S4 cells were grown in BG11 media supplemented with the same level of butanol stress (*i.e.*, 0.2%); and *ii*) Experiment II: S0, S2, S3, and S4 cells were grown in BG11 media supplemented with butanol equal to their maximal butanol tolerance levels for each cell sample (*i.e.*, 0.2, 0.25, 0.35 and 0.5% for S0, S2, S3, and S4, respectively). The rationale to establish two sets of experiments is that the effects of butanol concentration could be excluded when the results from the two experiments are carefully compared. The cells from both experiments were collected at middle exponential phase when they reach OD_730_ of 0.5 and subjected to LC-MS based metabolomic analyses. Each sample consisted three biological replicates and two technical replicates. After data normalization by the internal control and the cell numbers, two sets of the metabolomic profiles were analyzed separately by PCA plots (Figure 2A,B). The results showed that: *i*) the analysis has overall good reproducibility as variation between technical replicates were small (data now shown), and all three biological replicates tended to cluster together for each sample; *ii*) the analysis has overall good analytical resolution as a good separation between different sample clusters was clearly observed; *iii*) between the two analytical strategies, evolved cells subjected with the same 0.2% butanol (experiment I) or different concentrations of butanol equal to their maximal tolerance levels (experiment II), a very similar PCA plot pattern was observed: the starting cells without evolution (S0) was relatively separated from the cell samples collected from the later stages of the evolution courses, suggesting that once the adaptive evolution started, physiological changes in cells occurred quickly; in addition, the cells from the middle stages of the evolution course with a maximal butanol tolerance of 0.25 and 0.35% (S2 and S3) tended to cluster nearby, suggesting that at these stages, similar physiological changes probably occurred; moreover, the cells from later evolution course with maximal butanol tolerance of 0.5% (S4) was well separated from the cells of the two early evolution stages (S2 and S3). The PCA analysis also suggested an obvious pattern of gradual changes at the metabolite level during the adaptive evolution of butanol tolerance.

To further investigate the physiological changes during the evolution course, loading plots were generated to determine variation of individual metabolites for the above two LC-MS metabolomic experiments (Figure 2C,D). The score plot analysis showed that for the experiment I (*i.e.*, growth under 0.2% butanol), six potential biomarker metabolites, *D*-(−)-3-phosphoglyceric acid (3PG), *D*-fructose 6-phosphate (F6P), *D*-glucose 6-phosphate (G6P), NADPH, phosphoenolpyruvic acid (PEP) and *D*-ribose 5-phosphate (R5P), were found important for the discrimination of the cells from the four selected evolution stages (*i.e.*, maximal tolerance of 0.2, 0.25, 0.35 and 0.5% butanol, respectively) (Figure 2C); while for the experiment II (*i.e.*, growth under their maximal butanol tolerance levels), eight potential biomarker metabolites, ADP, F6P, G6P, *L*-glutamic acid (Glu), NADPH, 3PG, PEP and R5P, were found important for the discrimination of the S0, S2, S3 and S4 cells of the four evolution stages (Figure 2D). Interestingly, the two experiments shared very high similarity in terms of the discriminating metabolites identified, with six metabolites, F6P, G6P, NADP, 3PG, PEP and R5P, common for the both experiments, demonstrating that there were significant changes of the intracellular levels of these metabolites through the evolution course, and these metabolites could be key changes responsible for the improvement of butanol tolerance. Among them, NADPH is an important coenzyme participating in many cellular reactions, and their identification in tolerance-enhanced cells is also consistent with our previous proteomic analysis that found several NADPH-dependent enzymes, such as glycerol-3-phosphate dehydrogenase were responsive to exogenous butanol stress in *Synechocystis* [13]. Although most of other metabolites have never been reported for their roles in combating butanol stress, PEP as a glycolysis metabolite with a high-energy phosphate group has been reported with anti-oxidative properties [29], and responsive to osmotic stress in *Corynebacterium glutamicum* [30], while the changing levels of 3PG and R5P were found in plant under heat stress [31], and in *Saccharomyces cerevisiae* under acetic acid stress [32], respectively. In addition, ribose-5-phosphate isomerase that catalyzes the conversion between ribose-5-phosphate (R5P) and ribulose-5-phosphate (Ru5P) was recently found regulated under oxidative stress conditions in photosynthetic green algae *Chlamydomonas reinhardtii* [33]. Moreover, sucrose-phosphate synthase (SpsA, Sll0045) that uses F6P as substrate to form sucrose 6-phosphate is a key enzyme for synthesizing one major compatible solute, sucrose, against salt stress in *Synechocystis* [34-36]. Glucose-6-phosphate dehydrogenase that catalyzes the conversion from G6P and NADP to 6-phospho-*D*-glucono-1,5-lactone and NADPH, was responsive to radiation stress in *Synechococcus lividus* [37].

### GC-MS metabolomic analysis

In a previous study, we applied a GC-MS based metabolomic analysis to characterize the time-series metabolic responses of *Synechocystis* to butanol exposure, and the semi-quantitation analysis allowed identification of a dozen metabolites responsive to exogenous butanol stress [14]. In this study, the same GC-MS metabolomic analysis protocol was applied to the cells collected from the four selected evolution stages of butanol tolerance improvement. Following the similar strategies for LC-MS metabolomic analysis, S0, S1, S3, and S4 cells from four evolution stages across the evolution time course were selected (cells from day 0, 28, 205 and 350, corresponding to the wild type and the evolved strains with their maximal butanol tolerance of 0.2, 0.2, 0.35 and 0.5%, respectively) (Figure 1), and two cultivation experiments were conducted: *i*) Experiment I: S0, S1, S3, and S4 cells were grown in media supplemented with the same level of butanol stress (*i.e.*, 0.2%); and *ii*) Experiment II: S0, S1, S3, and S4 cells were grown in media supplemented with butanol equal to their maximal level of butanol tolerance for each sample (*i.e.*, 0.2, 0.2, 0.35 and 0.5%, respectively). As the LC-MS metabolomic analysis showed that S2 and S3 shared a very similar metabolic change (Figure 2), we selected S1 sample that is 44 days (11 passages) earlier than the S2 sample for the GC-MS metabolomic analysis. For each sample, three biological replicates were independently cultivated, metabolites-isolated and analyzed by GC-MS as described before [14,21]. Under the optimized analytical conditions, a good separation of intracellular metabolites was achieved on the GC column and further MS analysis allowed the chemical classification of a total 62 metabolites from *Synechocystis*, including various amino acids, sugars and organic acids, among which 55 and 48 metabolites were detected in all cells samples for experiment I and II, respectively (Additional file 2: Table S2, Additional file 3: Table S3).

**Figure 3:**
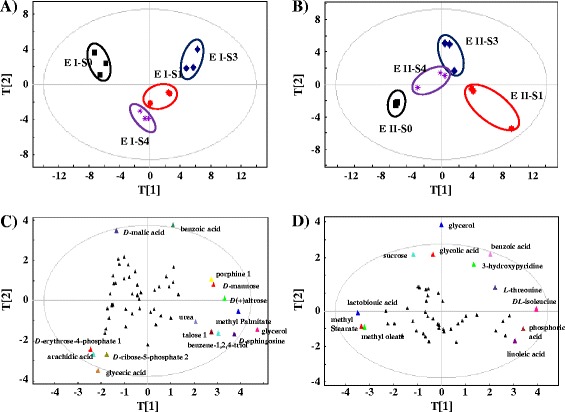
**PCA analysis of GC-MS metabolomic profiles. A)** Plot of the experiment I (S0, S1, S3 and S4 cells from day 0, 28, 205 and 350 of the evolution course, corresponding to their maximal butanol tolerance of 0.2, 0.2, 0.35 and 0.5%, respectively) grown in media supplemented with 0.2% (*v*/*v*) butanol; **B)** Plot of the experiment II (cells from day 0, 28, 205 and 350 of the evolution course, corresponding to their maximal butanol tolerance of 0.2, 0.2, 0.35 and 0.5%, respectively) grown in media supplemented with butanol equal to their maximal butanol tolerance levels, respectively; **C)** Loading plot of the experiment I; **D)** Loading plot of the experiment II.

PCA score plots were first applied to evaluate the similarities and differences between a total of 24 metabolomic profiles (Figure 3A,B). In general, the score plots of the GC-MS metabolomic profiles revealed the similar patterns as we described above for the LC-MS metabolomic profiles, such as overall good reproducibility between biological replicates and good separation between different sample clusters. In addition, the starting cells sample (S0) was also relatively separated from the cell samples collected from the later evolution stages. Moreover, the results showed that the profiles between early and middle evolution stages with a maximal butanol tolerance of 0.2 and 0.35% (*i.e.*, S1 and S3) were relatively separated in both experiments when compared with that between the S2 and S3 profiles used for LC-MS metabolomic analysis (Figure 3), suggesting greater metabolic difference between S1 and S3 than that between S2 and S3 used for LC-MS.

Loading plots were generated to determine variation of individual metabolites in the above two experiments (Figure 3C,D). The score plot analysis showed that for the experiment I (*i.e.*, growth under 0.2% butanol), the top potential biomarker metabolites that were important for the discrimination of the four evolutionary stages (*i.e.*, S0, S1, S3 and S4 cells, respectively) included *D*-(+) altrose, arachidic acid, benzoic acid, benzene-1,2,4-triol *D*-sphingosine, *D*-erythrose-4-phosphate glycerol, glyceric acid, *D*-malic acid, methyl palmitate, *D*-ribose-5-phosphate and talose (Figure 3C); while for the experiment II (*i.e.*, growth under their maximal butanol tolerance level), the top potential biomarker metabolites that were important for the discrimination of the S0, S1, S3 and S4 cells included benzoic acid, glycerol, glycolic acid, 3-hydroxypyridine, *DL*-isoleucine, lactobionic acid, linoleic acid, methyl oleate, methyl stearate, phosphoric acid, sucrose and *L*-threonine (Figure 3D). Two metabolites, glycerol and benzoic acid, were determined as discriminating metabolites in both experiments. Among all the major responsive metabolites during the evolution course of tolerance increase, glycerol, glyceric acid, 3-hydroxypyridine, *D*-malic acid, methyl palmitate, sucrose, talose and *L*-threonine were also previously identified as responsive to exogenous butanol in *Synechocystis* [14]. Some of them, such as sucrose, talose and threonine, although not reported for roles against butanol stress, have been found involved in responses to various environmental stresses in microbes [21,38].

### WGCNA analysis of metabolomic profiles associated with the elevated tolerance

**Figure 4:**
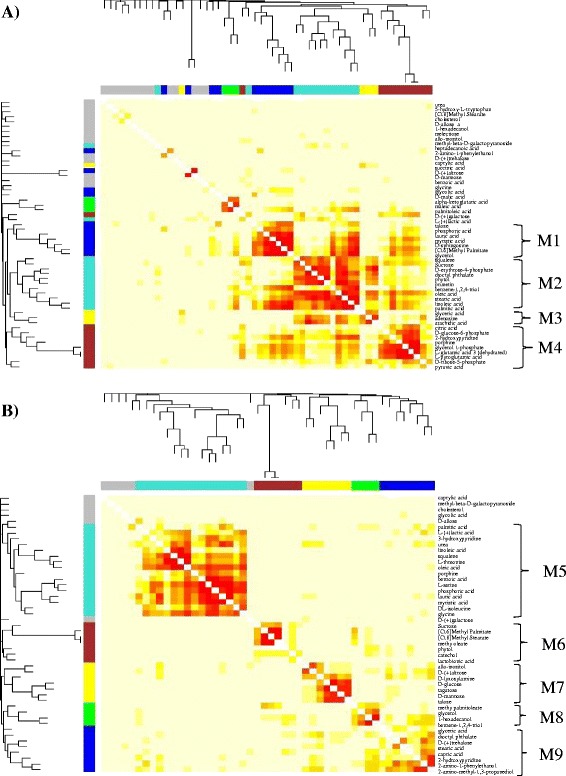
**Weighted Correlation Network Analysis (WGCNA) of GC-MS metabolic profiles of the** ***Synechocystis*** **during butanol tolerance improvement. A)** Experiment I; **B)** Experiment II. The distinct modules identified at each time point were indicted by the clustering patterns of the red color squares along the diagonal inside the plots. The modules highly associated with any given butanol stress (*r* > 0.5 and *p*-value <0.05) were identified and indicated by the color bar shown along the left side and at the top, where one color represents one distinct module. The metabolites associated with each of the distinct module were listed beside the plots with their module number indicated. The correlation coefficients and *p*-values were shown in the Tables 1 and 2.

To identify metabolic modules and hub metabolites related the gradual evolution of butanol tolerance, we also applied a WGCNA network analysis to the GC-MS metabolomic datasets. The analysis was not applied to LC-MS data due to their relatively small data size. WGCNA is a correlation-based and unsupervised computational method to describe and visualize correlation patterns of data points [39,40] and recently it was successfully applied to analyze metabolomic data from tomato [41] and *E. coli* and *Synechocystis* [21,42]. In this study, we compiled two separate GC-MS metabolomic datasets (*i.e.*, total 24 metabolomic profiles) consisted of 55 and 48 common metabolites identified in all samples for the experiment I and II, respectively. We then localized the correlated metabolites into various metabolic modules using the WGCNA approach for the two datasets. In addition, the association of each distinguished metabolic module with butanol stress or evolution stages was also determined, as highly associated modules indicated on the plots (Figure 4).

Setting a minimal number of metabolites in any module greater than 3, our WGCNA analysis showed that 6 distinct metabolic modules can be detected within the metabolic networks from both metabolomic datasets (Figure 4). The same number of metabolic modules detected from the experiments I and II was probably due to very similar physiological changes for the four cell samples along the evolution courses, even when they are cultivated under different concentration of butanol. Using a cutoff of correlation coefficient (*r* value) greater than 0.5 and their statistical confidence (*p*-values) less than 0.05, the analysis showed that a total of 4 and 5 distinguished metabolic modules were highly associated with butanol stress in the experiment I and II, respectively (Figure 4; Tables 1 and 2). Among them, 2 and 4 modules were uniquely associated with samples for the experiment I and II, respectively. These modules may represent the important metabolic changes during the gradual tolerance increase against butanol. In details, 1 and 2 distinguished metabolic modules were associated uniquely with the wild type strain under 0.20% butanol stress (S0) in experiment I and II, respectively; and 1 distinguished modules were associated uniquely with evolution stage S1 in the experiment II; 1 and 1 distinct modules were found associated uniquely with evolution stage S3 in the experiment I and II, respectively (Tables 1 and 2). Interestingly, the analysis showed that no highly associated metabolic module was detected in any of samples from the later evolution stage S4 (Figure 4).

**Table 1:** **Associated modules in experiment I**

**Module**	**Stage**	**Compound**	***r***	***p***
M1	S0	11	0.97	1.00E-07
M2	S0	13	−0.64	0.02
	S3	13	0.92	2.00E-07
M3	S3	4	0.67	0.02
M4	S0	10	−0.7	0.01
	S1	10	0.58	0.05

**Table 2:** **Associated modules in experiment II**

**Module**	**Stage**	**Compound**	***r***	***p***
M5	S1	16	0.86	4.00E-04
M6	S3	7	0.83	8.00E-04
M7	S0	7	−0.7	0.01
M8	S0	4	−0.71	0.01
M9	S0	8	−0.83	8.00E-04
	S1	8	0.57	0.05

Analysis of the constitute of the modules showed that module M1 positively associated with S0 sample contained glycerol and short chain (C12 ~ 16) fatty acid such as lauric acid and myristic acid, while module M7 and M8 negatively associated with S0 sample contained allo-inositol, *D*-(+) altrose, *D*-lyxosylamine, *D*-glucose, tagatose, *D*-mannose, talose, methy palmitoleate, glycerol,1-hexadecanol, and benzene-1,2,4-triol; module M5 positively associated with S1 sample contained 4 amino acids (*i.e.*, serine, isoleucine, glycine and threonine), 6 fatty acids (*i.e.*, palmitic acid, lauric acid, myristic acid, squalene, linoleic acid and oleic acid), urea and lactic acid; module M3 positively associated with S3 sample contained glyceric acid, adenosine and arachidic acid; module M6 positively associated with S3 sample contained sucrose, methyl palmitate, methyl stearate, methy oleate, phytol, catechol, and lactobionic acid.

**Figure 5:**
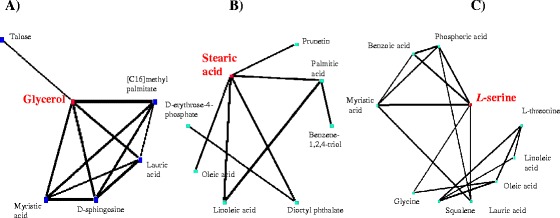
**Hub metabolites and their metabolic profile as represented by node and edge graph. A)** glycerol in module M1; **B)** stearic acid in module M2; **C)** L-serine in module M5.

Hubs are genes or metabolites with high degree of connectivity in biological interaction networks and are thus supposed with high biological importance [43]. Most hubs in natural networks such as ATP, NADH, glutamate, and coenzyme A are key compounds in the transfer of biochemical groups in the networks [44]. Within the metabolic network constructed by the WGCNA approach, assuming a cutoff of connectivity greater than 5 in the networks as hub metabolites, we were able to identified three hub metabolites, glycerol, stearic acid and serine, associated with the butanol-responsive modules of M1, M2, and M5, respectively (Figure 5). The first hub metabolite, glycerol, was connected with talose, sphingosin, methyl palmitate and several short chain fatty acids, such as lauric acid and myristic acid (Figure 5A). Glycerol synthesis is associated with the regeneration of oxidized cofactors (NAD^+^), playing a role in the control of the redox balance [45], and the elevated production of glycerol by yeast was also observed under osmotic stress conditions [46] and adaptation to ethanol stress in yeast [47]. In yeast, it has been suggested that most yeasts rapidly produce glycerol under ethanol stress as an alternative means of NAD^+^ regeneration rather than having a specific requirement for glycerol [47]. Interestingly, the second hub metabolite, stearic acid (C18), was tightly connected with several other fatty acids, such as palmitic acid (C16 saturated), oleic acid and linoleic acid (C18, unsaturated), and with benzene-1,2,4-triol, dioctylphthalate and erythrose-4- phosphate (Figure 5B). The third hub metabolite, serine, was tightly connected with several amino acids (*i.e.*, glycine and threonine) and some fatty acids (myristic acid, linoleic acid, oleic acid, lauric acid, and squalene) (Figure 5C). The results suggested that amino acids and fatty acids could be the key protection mechanisms against butanol stresses. It was previously reported that amino acids could be involved in stress resistance to acid and various biofuel products in *E. coli* [48-50] and in response to long-term salt stress in *Synechocystis* [42]. Role of lipids and fatty acids in stress tolerance in bacteria has been well-documented, *i.e.*, the control of membrane fluidity during the heat-shock response can be accounted for, at least in part, by the changes in the fatty acid composition of *E. coli* [51]. In addition, alternations of lipids and fatty acids responding to various environmental or salt stresses were also reported in cyanobacteria [52,53].

## Conclusions

Toxic effects of biofuels to microbes have been considered as one major hurdle for high-efficiency biofuel production [10-12,54]. To obtain butanol-tolerant cyanobacterial strains, we performed a laboratory-based evolution by growing *Synechocystis* under gradually increased butanol stress. After an adaptive evolution process of 94 passages or 395 days under butanol selective pressure, the butanol tolerance of *Synechocystis* was improved by 150%. To further explore the mechanism responsible for the tolerance increase, we applied an integrated LC-MS and GC-MS based metabolomic analyses to determine the variation of both unstable and stable intracellular intermediates across the evolution time course. In addition, a WGCNA network analysis was applied the metabolomic datasets to reveal the responsive metabolic modules and key hub metabolites through the evolution course. Due to high complexities of the cells, cellular networks are typically organized into various functional modules that can be individually controlled by different regulatory proteins, as a recent study showed that overexpression of a sigma factor SigB in *Synechocystis* resulted in increased tolerance to temperature and butanol [55]. The determination of the metabolic modules related to butanol tolerance in this study may thus represent the first step in defining their regulators and further transcriptional engineering to improve tolerance to butanol. The study provided the first time-series description of the metabolomic changes related to the gradual increase of butanol tolerance, and revealed metabolomic basis important for further rational engineering in *Synechocystis* [56], which also highlights the values in applying integrated LC-MS and GC-MS in fully deciphering microbial metabolism. By integrating the metabolomic information with various genetic functional genomic analyses of the evolved strains, once they are available in the future, will significantly improve our understanding of the butanol tolerance in cyanobacteria. Finally, in this study the metabolomic profiles of the evolved *Synechocystis* strains were determined with butanol supplied extracellularily, it will be interesting if a engineered butanol-producing *Synechocystis*, once available, can also be analyzed by the similar strategy, and the metabolomic basis against intracellular butanol can be compared with the results obtained form this study, which will be very helpful in further deciphering the tolerance mechanism of butanol in *Synechocystis*.

## Materials and methods

### Bacterial growth conditions

*Synechocystis* sp. PCC 6803 and the laboratory-evolved mutants were grown in BG11 medium (pH 7.5) under a light intensity of approximately 50 μmol photons m^−2^ s^−1^ in an illuminating incubator of 130 rpm at 30°C (HNY-211B Illuminating Shaker, Honour, China) [13,14]. Cell density was measured on a UV-1750 spectrophotometer (Shimadzu, Japan).

### Experimental evolution of butanol tolerance

Butanol of analytical pure was purchased from Merck (U.S.A.). Experimental evolution of butanol tolerance was conducted by serial passaging four independent *Synechocystis* populations on liquid BG11 media supplemented with butanol. Cultures were grown in 250-mL flasks containing 50 mL BG11 medium amended with varying concentration of butanol. The initial butanol concentration was 0.2% (*v*/*v*), and was gradually increased to 0.5% (*v*/*v*) during the experimental evolution process. Cultures were passaged when populations reached middle exponential phase with OD_730_ of 0.5 (typically from 3–5 days). Butanol concentration was increased by 0.05% when the culture reached OD_730_ of 0.5 within three days. Each lineage was periodically checked for contamination by observing using a BX43 fluorescence microscope (Olympus, Japan) and isolation streaking culture samples on BG11 agar plates. Samples from each population were cryopreserved every 5 passages by centrifuging 2.0 mL culture at 8,000 × *g* for 5 min, washing the cell pellets with fresh BG11 medium, centrifuging again, and resuspending cell pellets in 150 μL fresh medium with 15% (*v*/*v*) glycerol, and stored at −80°C. The experimental evolution proceeded for 94 passages or 395 days, corresponding to approximately ~700 generations, assuming an average ~7.5 generations per passage based on previous estimation of the growth rate of *Synechocystis* [22,23].

### LC-MS based metabolomics analysis

*i*) *Sample quenching, extraction, and preparation*: All chemicals used for LC-MS metabolomic analyses were obtained from Sigma-Aldrich (Taufkirchen, Germany). Cells were collected by centrifugation at 8,000 × *g* for 8 min at room temperature (Eppendorf 5430R, Hamburg, Germany). The cell samples were quenched and extracted rapidly with 900 μL of 80:20 MeOH/H_2_O (−80°C) and then frozen in liquid nitrogen. The samples were then frozen-thawed three times to release metabolites from the cells. The supernatant was collected after centrifugation at 15,000 × *g* for 5 min at −4°C and then stored at −80°C. The remaining cell pellets were re-suspended in 500 μL of 80:20 MeOH/H_2_O (−80°C) and the above extraction process was repeated. The supernatant from the second extraction was pooled with that from the first extraction and stored at −80°C until LC-MS analysis [27]; *ii*) *LC-MS analysis*: The chromatographic separation was achieved with a SYnergi Hydro-RP (C18) 150 mm × 2.0 mm I.D., 4 μm 80 Å particles column (Phenomenex, Torrance, CA, USA) at 40°C. Mobile phase A (MPA) is an aqueous 10 mM tributylamine solution with pH 4.95 adjusted with acetic acid and Mobile phase B (MPB) is 100% methanol of HPLC grade (Darmstadt, Germany). The optimized gradient profile was determined as follows: 0 min (0% B), 8 min (35% B), 18 min (35% B), 24 min (90% B), 28 min (90% B), 30 min (50% B), 31 min (0% B). A 14-minute post-time equilibration was employed, bringing total run-time to 45 min. Flow rate was set as a constant 0.2 mL/min [57]. LC-MS analysis was conducted on an Agilent 1260 series binary HPLC system (Agilent Technologies, Waldbronn, Germany) coupled to an Agilent 6410 triple quadrupole mass analyser equipped with an electrospray ionization (ESI) source. Injected sample volume for all cases was 10 μL; capillary voltage was 4000 V; and nebulizer gas flow rate and pressure were 10 L/min and 50 psi, respectively. Nitrogen nebulizer gas temperature was 300°C. The MS was operated in negative mode for multiple reaction monitoring (MRM) development, method optimization, and sample analysis. Data were acquired using Agilent Mass Hunter workstation LC/QQQ acquisition software (version B.04.01) and chromatographic peaks were subsequently integrated *via* Agilent Qualitative Analysis software (version B.04.00); *iii*) *Targeted metabolite analysis*: a total of 24 metabolites were selected for LC-MS based targeted metabolite analysis in this study. The abbreviations, molecular weights and MRM values determined and optimized for each of the 24 detected metabolites as well as the product ion formulas were provided in Additional file 1: Table S1. The standard compounds for these 24 metabolites were purchased from Sigma, and their MS and MS/MS experimental parameters were optimized with the mix standard solution. All metabolomics profile data was first normalized by the internal control and the cell numbers of the samples, and then subjected to Principal Component Analysis using software SIMCA-P 11.5 [58].

### GC-MS based metabolomics analysis

All chemicals used for metabolome isolation and GC-MS analyses were obtained from Sigma-Aldrich (Taufkirchen, Germany). For GC-MS metabolomic analysis, cells were collected by centrifugation at 8,000 × *g* for 8 min at 4°C (Eppendorf 5430R, Hamburg, Germany). The cell pellets were immediately frozen in liquid nitrogen and then stored at −80°C before use. The metabolomic analysis protocol included: *i*) *Metabolome extraction*: cells were re-suspended in 1.0 mL cold 10:3:1 (*v*/*v*/*v*) methanol:chloroform:H_2_O solution (MCW), and frozen in liquid nitrogen and thawed for five times. Supernatants were collected by centrifugation at 15,000 × *g* for 3 min at 4°C (Eppendorf 5430R, Hamburg, Germany). To normalize variations across samples, an internal standard (IS) solution (100 μg/mL U-13C-sorbitol,10 μL) was added to 100 μL supernatant in a 1.5-mL microtube before it was dried by vacuum centrifugation for 2–3 h (4°C). *ii*) *Sample derivatization*: derivatization was conducted according to the two-stage technique by Roessner et al. [59]. The samples were dissolved in 10 μL methoxyamine hydrochloride (40 mg/mL in pyridine) and shaken at 30°C for 90 min, then were added with 90 μL N-methyl-N-(trimethylsilyl) trifluoroacetamide (MSTFA) and incubated at 37°C for 30 min to trimethylsilylate the polar functional groups. The derivate samples were collected by centrifugation at 15,000 × *g* for 3 min before GC/MS analysis. *iii*) *GC-MS analysis*: analysis was performed on a GC-MS system-GC 7890 coupled to an MSD 5975 (Agilent Technologies, Inc., Santa Clara, CA, USA) equipped with a HP-5MS capillary column (30 m × 250 mm id). 2 μL derivatized sample was injected in splitless mode at 230°C injector temperature. The GC was operated at constant flow of 1 mL/min helium. The temperature program started isocratic at 45°C for 2 min, followed by temperature ramping of 5°C/min to a final temperature of 280°C, and then held constant for additional 2 min. The range of mass scan was m/z 38–650. *iv*) *Data processing and statistical analysis*: The mass fragmentation spectrum was analyzed using the Automated Mass Spectral Deconvolution and Identification System (AMDIS) [60] to identify the compounds by matching the data with Fiehn Library [61] and the mass spectral library of the National Institute of Standards and Technology (NIST). Peak areas of all identified metabolites were normalized against the internal standard and the acquired relative abundances for each identified metabolite were used for future data analysis. All metabolomics profile data was first normalized by the internal control and the cell numbers of the samples, and then subjected to PCA analysis using software SIMCA-P 11.5 [58].

### WGCNA network construction

Correlation network was created from the GC-MS metabolomic data, first by calculating weighted *Pearson* correlation matrices corresponding to metabolite abundance, and then by following the standard procedure of WGCNA to create the networks [39-41,49]. Briefly, weighted correlation matrices were transformed into matrices of connection strengths using a power function [41]. These connection strengths were then used to calculate topological overlap (TO), a robust and biologically meaningful measurement that encapsulates the similarity of two metabolites’ correlation relationships with all other metabolites in the network [41]. Hierarchical clustering based on TO was used to group metabolites with highly similar correlation relationships into modules. Metabolite dendrograms were obtained by average linkage hierarchical clustering [40,41,50], while the color row underneath the dendgram showed the module assignment determined by the Dynamic Tree Cut of WGCNA. The network for each module was generated with the minimum spanning tree with dissimilarity matrix from WGCNA. The modules with correlation *r* >0.5, and *p*-value less than 0.05 were extracted for further investigation. Hub metabolites were screened by high connectivity with other metabolites (≥5) in the modules strongly associated with phenotype (each of biofuel treatments, based on correlation coefficient *r* >0.5).

## Additional files

Additional file 1: Table S1. Compound-specific collisional mass spectrometric parameters used in MRM. Additional file 2: Table S2. GC-MS metabolomic dataset from the experiment I. Additional file 3: Table S3. GC-MS metabolomic dataset for the experiment II.

## Abbreviations

GC-MS: Gas chromatography–mass spectrometry
LC-MS: Liquid Chromatography–Mass Spectrometry
PCA: Principal Component Analysis
WGCNA: Weighted Correlation Network Analysis
