# Supplementary material for: Metabolomic basis of laboratory evolution of butanol tolerance in photosynthetic Synechocystis sp. PCC 6803
Source: Microb Cell Fact. 2014 Nov 1;13:151. doi: 10.1186/s12934-014-0151-y (PMC4234862; doi:10.1186/s12934-014-0151-y)
Supplement: Additional file 1: Table S1. — Compound-specific collisional mass spectrometric parameters used in MRM. [file 12934_2014_151_MOESM1_ESM.pdf]

Supplementary Table S1. Compound-specific collisional mass spectrometric parameters used in MRM.

Compound	Abbrev	MW[1]	$(M-H)^-$	FV[2](V)	Product ion	Product fromula	CV[3](V)
Acetyl coenzyme A	AcCOA	809.57	808	260	79	$[PO_3]^-$	130
NADPH	NADPH	833.35	744	200	79	$[PO_3]^-$	112
NADP	NADP	787.37	742	140	620	-nicotinamide	8
NADH	NADH	709.4	664	190	79	$[PO_3]^-$	108
α-Nicotinamide adenine dinucleotide	NAD	685.41	662	100	540	-nicotinamide	8
Adenosine-5'-diphosphoglucose	ADP-GCS	633.31	588	170	346	AMP	20
Uridine 5'-diphosphoglucose	UDP-GCS	610.27	565	160	323	UMP	10
Adenosine 5'-triphosphate (ATP)	ATP	551.14	506	142	79	$[PO_3]^-$	92
Adenosine 5'-diphosphate	ADP	501.32	426	150	79	$[PO_3]^-$	60
Coenzyme A hydrate	COA	767.53	382.5	110	79	$[PO_3]^-$	68
Adenosine 5'-monophosphate	AMP	347.22	346	140	79	$[PO_3]^-$	45
D-Fructose 1,6-bisphosphate	FBP	406.06	339	110	97	$[H_2PO_4]^-$	18
D-Ribulose 1,5-bisphosphate	RiBP	310.09	309	104	97	$[H_2PO_4]^-$	14
D-Fructose 6-phosphate	F6P	304.1	259	86	97	$[H_2PO_4]^-$	4
D-Glucose 6-phosphate	G6P	304.1	259	100	97	$[H_2PO_4]^-$	4
D-Ribose 5-phosphate	R5P	310.1	229	78	97	$[H_2PO_4]^-$	12
D-(−)-3-Phosphoglyceric acid	3PG	230.02	185	84	97	$[H_2PO_4]^-$	4
Dihydroxyacetone phosphate	DHAP	180.19	169	84	97	$[H_2PO_4]^-$	4
DL-Glyceraldehyde 3-phosphate	GAP	170.06	169	66	97	$[H_2PO_4]^-$	8
Phospho(enol)pyruvic acid	PEP	190.02	167	82	79	$[PO_3]^-$	4
L-Glutamic acid	GLU	187.13	145	80	127	$[C_5H_8N_2O_2]^-$	8
α-Ketoglutaric acid	AKG	226.09	145	60	101	$[C_4H_5O_3]^-$	6
Oxaloacetic acid	OXA	132.07	131	110	87	$[C_3H_3O_3]^-$	0
Sodium fumarate dibasic	FUM	160.04	115	58	71	$[C_3H_3O_2]^-$	0

[1] Molecular weight

[2] Fragmentation voltage at which maximal parent ion intensity is observed

[3] Collision cell voltage at which maximal product ion intensity is observed
