# Supplementary material for: Metabolomic basis of laboratory evolution of butanol tolerance in photosynthetic Synechocystis sp. PCC 6803
Source: Microb Cell Fact. 2014 Nov 1;13:151. doi: 10.1186/s12934-014-0151-y (PMC4234862; doi:10.1186/s12934-014-0151-y)
Supplement: Additional file 2: Table S2. — GC-MS metabolomic dataset from the experiment I. [file 12934_2014_151_MOESM2_ESM.pdf]

Supplementary Table S2. GC-MS metabolomic dataset from the experiment I. A, B, and C represent biological triplicates for each treatment.

Metabolite	S0A	S0B	S0C	S1A	S1B	S1C	S3A	S3B	S3C	S4A	S4B	S4C
[C16]Methyl-Palmitate	0.0039764	0.0054525	0.00352	0.0005703	0.0009432	0.0002725	0.0004385	0.0007737	0.0005874	0.000263	0.000714	0.0015377
[C18]Methyl-Stearate	0.0004863	0.0019605	0.0004599	0.0006441	4.777E-05	0.0003953	4.253E-05	0.0024807	0.0001647	0.0003006	3.248E-05	0.0005889
1-hexadecanol	5.234E-05	3.388E-05	1.655E-05	0.001704	0.0005459	0.0002378	0.0002776	0.0004071	0.000105	0.0006734	0.0005471	0.0003025
2-amino-1-phenylethanol	0.0008313	0.0007365	0.0004236	0.0004069	0.003762	0.005225	0.0005434	0.0016879	0.0004685	0.0005115	0.0006466	0.0019776
2-hydroxypyridine	0.0014859	0.0020408	0.0016245	0.0040894	0.0029642	0.0034843	0.0033311	0.0047818	0.0034528	0.0026006	0.0034286	0.0029451
5-hydroxy-L-tryptophan	0.0001367	0.000113	0.000186	3.808E-05	0.000148	9.035E-05	2.216E-05	0.0003844	0.0002292	7.463E-05	2.47E-05	9.588E-05
adenosine	0.0012486	0.000584	5.449E-05	0.0005431	0.0018425	0.0023413	0.004041	0.0029711	0.0018808	0.0013135	0.0005524	0.0012856
allo-inositol	7.759E-05	0.0001274	0.000205	7.73E-05	0.0001539	0.0001451	4.742E-05	7.177E-05	0.0001474	0.0002289	0.00019	0.0001109
alpha-ketoglutaric-acid	0	0	0	7.668E-05	0.0001176	5.729E-05	1.142E-05	0.0005963	8.124E-05	0.0001023	0.0011411	7.564E-05
arachidic-acid	0	0	0	4.363E-05	8.821E-05	0.0003493	0.002292	0.001877	0.0003392	2.019E-05	8.94E-05	5.301E-05
benzene-1,2,4-triol	0.000303	0.0002508	0.000236	0.0001738	6.236E-05	0.0002196	2.186E-05	1.333E-05	5.806E-06	2.765E-05	0	0
benzoic-acid	0.0151216	0.0106274	0.0241274	0.0049777	0.0063964	0.0151193	0.0067426	0.0082775	0.0102556	0.0427246	0.0636741	0.0741493
caprylic-acid	4.429E-06	7.563E-06	7.412E-06	0	0.0001766	0.0001564	0.0002165	8.306E-06	0	0.0001791	7.34E-05	0.0001227
cholesterol	0	0.0008923	0	5.101E-05	1.663E-05	5.535E-05	0.0005248	0.0005451	0.00029	0.0002632	6.261E-05	0.0001436
citric-acid	0	0	0	0.0003234	0	0.0003468	4.946E-05	0.0003923	6.675E-05	0.0001693	4.413E-05	0
D-(+)-galactose	0.0001453	0.0002048	0.0001008	0.0006987	9.292E-05	0.0004665	0.0002855	0.0004327	0.0003917	5.314E-05	3.578E-05	1.529E-05
D-(+)-altrose	0.0001636	0.0008916	9.154E-05	1.739E-05	0.0005525	0	3.009E-05	0	3.764E-05	0	9.347E-05	0.0002043
D-(+)-trehalose	0.0002706	0.0001687	5.647E-05	7.557E-05	0.0002383	0.0002531	0.0004845	0.0005459	0.0005869	0.0003554	0.0004422	0.0001697
D-allose	0.0001453	0.0010463	0.0001115	0	0.0005414	0.0004665	0.0002855	0.0007475	0.0004277	0.0006915	0.0004752	0.0004009
D-erythrose-4-phosphate	0	0	0	1.775E-05	3.064E-05	9.429E-05	0.0009169	0.000581	0.0005938	0	0	2.539E-06
D-glucose-6-phosphate	0	0	0	0.0026895	0.00334	0.0021725	0.0014914	0.0026504	0.0032562	0	0	5.033E-06
dioctyl-phthalate	0.0013074	0.0016961	0.0012289	0.0015421	0.0014597	0.0012642	0.0039071	0.0043268	0.0038354	0.0020243	0.0021037	0.0018389
D-malic-acid	0	0	0	0.0009826	7.215E-05	0.0002803	0.0005189	1.437E-05	0.0006936	0.0016834	0.0004775	0.0005249
D-mannose	0.0002378	0.0006316	0.0001008	0	0.0005525	6.519E-05	7.757E-06	7.423E-05	7.396E-05	0	8.29E-05	0.000451
D-ribose-5-phosphate	0	0	0	8.546E-05	0.0007379	0.0010689	0.0007671	0.0012008	0.0002704	0	0	0
D-sphingosine	0.0028671	0.0033735	0.0035925	0.0004891	0.0002354	0.0005989	0.0006214	0.0008192	0.0005935	2.766E-05	0.0005347	1.382E-05
glyceric-acid	0	0	0	0	0.0001093	0.0303377	0.0512237	0.0092952	9.992E-06	0	0	2.501E-05
glycerol	0.2415517	0.2589466	0.1757083	0.0165967	0.0352942	0.0458216	0.0106583	0.010028	0.0091337	0.0122686	0.0118093	0.0147777
glycerol-1-phosphate	0	0	0	0.0030215	0.0023272	0.0040829	0.002823	0.0034366	0.0024494	0.0013393	0.0011989	0.0017248
glycine	0.0002333	0.0006119	0.0002647	0.0002329	0.0002453	0.0004345	0.0001898	0.0001414	3.092E-05	0.0010523	0.0002147	0.000335
glycolic-acid	0.0360998	0.0518875	0.0330327	0.0205515	0.0142526	0.0223124	0.0273697	0.0286738	0.0197487	0.045295	0.0094941	0.0196776
heptadecanoic-acid	0.0001232	0.0001396	0.0001078	6.072E-05	0	4.168E-05	0.0001636	0.0001441	0.0003	0.0001527	0.0001288	9.279E-05
L-(+)-lactic-acid	0.036191	0.0463051	0.0297873	0.0205515	0.0433429	0.0281646	0.0273697	0.0286738	0.0199933	0.045295	0.0359523	0.038958
lauric-acid	8.524E-06	1.895E-05	1.565E-05	0.0005999	0.0006343	0.000714	0.0004868	0.0007778	0.0004276	0.0007923	0.0005364	0.0010329
L-glutamic-acid-3-(dehydrated)	5.14E-05	2.051E-05	0	0.0159023	0.0103849	0.0166024	0.0116071	0.0136054	0.0102404	0.0054573	0.0054304	0.0061809
linoleic-acid	0	0	0	0.0026266	0.0020545	0.0016848	0.0030183	0.0030934	0.0039822	0.0024349	0.0019457	0.0019131
L-pyroglutamic-acid	0.0001123	0.0001308	9.224E-05	0.017968	0.0116843	0.0170288	0.0116071	0.0136054	0.0102439	0.0071361	0.000684	0.0069162
maleic-acid	0	0	0	5.173E-06	7.548E-06	9.122E-05	0.000268	0.0003343	9.357E-05	0.0003026	0.0005025	0.0001181

melezitose	0.0001088	0.0001687	7.722E-05	0.0001873	0.0002317	0.0003715	7.066E-05	0.0002142	0.0008555	0.0002155	0.0003078	0.000126
methyl-beta-D-galactopyranoside	0.000708	0.0009134	0.0014717	0.0005703	0.0028303	6.458E-05	0.0003888	0.0009004	0.000298	0.0018838	0.0014988	0.0010985
myristic-acid	0.0002785	7.231E-05	5.307E-05	0.0022471	0.0017607	0.0027228	0.0018681	0.0018341	0.0028532	0.0017403	0.0022602	0.0023977
oleic-acid	0	0	0	0.0019653	0.0017042	0.0021483	0.0035938	0.004362	0.0032626	0.0014689	0.0007859	0.0012708
palmitic-acid	0.0002296	0.0001475	0.0002188	0.1103709	0.1061943	0.1012076	0.1767734	0.1859701	0.1905699	0.1334102	0.1354994	0.1144688
palmitoleic-acid	0	0	0	0.0009694	0.0004103	0.0003972	0.0012213	0.001498	0.0009279	0.0008791	0.0020412	0.0003934
phosphoric-acid	0.0003364	0.0001884	0.004448	0.2221309	0.3573119	0.4282986	0.1497683	0.1317047	0.1418743	0.1867737	0.2504924	0.2934302
phytol	0.0015121	0.0006489	0.0008208	0.001653	0.0010436	0.0014657	0.0026668	0.0025727	0.0028891	0.000849	0.0011558	0.0011051
porphine	0.166477	0.1728083	0.1655553	0.0158802	0.0213429	0.0303377	0.0525867	0.0475152	0.0401127	0.03722	0.1536431	0.1833836
prunetin	1.473E-05	1.319E-05	2.072E-05	8.499E-05	0.0001265	9.315E-05	0.0002658	0.0002652	0.0004076	5.371E-05	2.048E-05	6.363E-05
pyruvic-acid	0	0	0	0.0016758	0.0021911	0.0017032	0.0006251	0.0059008	0.0002494	0.0003955	0.0002732	0.0001637
squalene	0.0007979	0.0005157	0.0001066	0.0018421	0.0009197	0.0013469	0.0025995	0.0016824	0.0018744	0.0007767	0.0011543	0.0003723
stearic-acid	0.0008543	0.0008467	0.0005525	0.0847905	0.0832435	0.0630915	0.1947983	0.2258281	0.2106397	0.1053034	0.1002837	0.0833617
succinic-acid	7.005E-05	0.0001776	0.0001087	0.0002932	0.0001816	0.0004788	0.0012169	0.0004436	8.172E-05	0.0001855	0.0005917	0.0008083
sucrose	0.0231378	0.0175962	0.0214683	0.114068	0.0537618	0.0657622	0.1348315	0.149954	0.107009	0.0047805	0.0036514	0.0030721
talose	0.000708	0.0007927	0.0005765	0	0.0002267	0.0004665	0	0.0006617	7.988E-05	0	8.29E-05	0.0002221
urea	7.251E-05	0.0002139	0.0015832	0	3.258E-05	0.0010528	1.715E-05	0.0001805	1.878E-05	0.0003134	0.000258	0.000151
