# Supplementary material for: Metabolomic basis of laboratory evolution of butanol tolerance in photosynthetic Synechocystis sp. PCC 6803
Source: Microb Cell Fact. 2014 Nov 1;13:151. doi: 10.1186/s12934-014-0151-y (PMC4234862; doi:10.1186/s12934-014-0151-y)
Supplement: Additional file 3: Table S3. — GC-MS metabolomic dataset for the experiment II. [file 12934_2014_151_MOESM3_ESM.pdf]

**Supplementary Table S3. GC-MS metabolomic dataset for the experiment II. A, B, and C represent biological triplicates for each treatment.**

| Metabolite | S0A | S0B | S0C | S1A | S1B | S1C | S3A | S3B | S3C | S4A | S4B | S4C |
|---|---|---|---|---|---|---|---|---|---|---|---|---|
| [C16]Methyl-Palmitate | 0.0039764 | 0.0054525 | 0.00352 | 0.009978 | 0.0033722 | 0.0086439 | 0.0170071 | 0.0309878 | 0.0381051 | 0.0103939 | 0.0211449 | 0.0185467 |
| [C18]Methyl-Stearate | 0.0004863 | 0.0019605 | 0.0004599 | 0 | 0.0006117 | 0.0008022 | 0.0187921 | 0.0224009 | 0.039835 | 0.0059899 | 0.0135635 | 0.0114643 |
| 1-hexadecanol | 5.23E-05 | 3.39E-05 | 1.65E-05 | 0.0002638 | 0.0002042 | 0.0002 | 0.000229 | 0.0004019 | 0.0002571 | 5.99E-05 | 0.0002074 | 0.0001058 |
| 2-amino-1-phenylethanol | 0.0008313 | 0.0007365 | 0.0004236 | 0.03752 | 0.0226432 | 0.0190905 | 0.0141998 | 0.0222626 | 0.0159626 | 0.014205 | 0.012442 | 0.0067538 |
| 2-amino-2-methyl-1,3-propanediol | 0.0001603 | 0.0002053 | 0.0001374 | 0.001345 | 0.0012684 | 0.0010543 | 0.0010892 | 0.0012071 | 0.0006794 | 0.0011233 | 0.0008256 | 0.0004297 |
| 2-hydroxypyridine | 0.0014859 | 0.0020408 | 0.0016245 | 0.0039169 | 0.0039415 | 0.0041769 | 0.0040014 | 0.0040797 | 0.0017626 | 0.0034534 | 0.0041755 | 0.0030266 |
| 3-hydroxypyridine | 0.0001309 | 0.0002036 | 5.39E-05 | 0.0002633 | 0.000361 | 0.0001744 | 3.99E-05 | 0.0001519 | 0.000143 | 1.83E-05 | 2.23E-05 | 1.114E-05 |
| allo-inositol | 7.76E-05 | 0.0001274 | 0.000205 | 0.0003659 | 0.0005206 | 0.0004883 | 0.0005164 | 0.0001321 | 0.0005042 | 0.0005386 | 0.0004964 | 0.0003257 |
| benzene-1,2,4-triol | 0.000303 | 0.0002508 | 0.000236 | 0.0007712 | 0.0006927 | 0.0009176 | 0.0006895 | 0.0009171 | 0.0006699 | 0.000248 | 0.0007849 | 0.0004634 |
| benzoic-acid | 0.0151216 | 0.0106274 | 0.0241274 | 0.0054989 | 0.0824141 | 0.0029035 | 0.0100983 | 0.0033225 | 0.0019362 | 0.0020867 | 0.006093 | 0.0031294 |
| capric-acid | 0.000291 | 0.0001184 | 0.0002504 | 0.0027564 | 0.0008195 | 0.0031801 | 0.0014213 | 0.0023275 | 0.002085 | 0.0019855 | 0.000999 | 0.0005199 |
| caprylic-acid | 4.43E-06 | 7.56E-06 | 7.41E-06 | 0.000101 | 0.0001593 | 1.47E-05 | 9.21E-05 | 0 | 0 | 0.0003525 | 0.0006334 | 0.0003349 |
| catechol | 7.00E-05 | 0.0001369 | 0.0001087 | 0.0001679 | 3.11E-05 | 0.0002133 | 0.0001382 | 0.0003676 | 0.0002295 | 0.0001619 | 0.000148 | 7.818E-05 |
| cholesterol | 0 | 0.0008923 | 0 | 6.04E-05 | 0.0005947 | 0.0007923 | 0.000807 | 0.0008453 | 0.0005752 | 0.0002879 | 0.0003409 | 0.0001704 |
| D-(+)galactose | 0.0001453 | 0.0002048 | 0.0001008 | 0.009884 | 0.0045015 | 0.0193544 | 0.0186956 | 0.0017388 | 0.0259214 | 0.0054815 | 0.0028015 | 0.0039236 |
| D-(+)altrose | 0.0001636 | 0.0008916 | 9.15E-05 | 0.0021115 | 0.003771 | 0.0024428 | 0.0028505 | 0.0023279 | 0.0034222 | 0.0029817 | 0.0034388 | 0.001963 |
| D-(+)trehalose | 0.0002706 | 0.0001687 | 5.65E-05 | 0.0037719 | 0.0016252 | 0.0010657 | 0.0009302 | 0.0009171 | 0.0021272 | 0.002459 | 0.001235 | 0.0008552 |
| D-allose | 0.0001453 | 0.0010463 | 0.0001115 | 0.0007996 | 0.003076 | 0.0022069 | 0.0054495 | 0.0004752 | 0.0016258 | 0.0001692 | 0.000273 | 0.0032842 |
| D-glucose | 0.0002378 | 0.0002947 | 0.0001008 | 0.0023342 | 0.0228879 | 0.0222192 | 0.0037369 | 0.0023279 | 0.0042793 | 0.0539323 | 0.0034388 | 0.0042423 |
| dioctyl-phthalate | 0.0013074 | 0.0016961 | 0.0012289 | 0.0019661 | 0.0029164 | 0.002749 | 0.0025093 | 0.0036061 | 0.0025792 | 0.0031476 | 0.0022826 | 0.00134 |
| DL-isoleucine | 0 | 0 | 7.81E-05 | 0.0001757 | 0.000184 | 3.88E-05 | 0 | 0 | 0 | 0 | 0 | 6.205E-06 |
| D-lyxosylamine | 0 | 0 | 0 | 0.000258 | 0.0004661 | 0.0002327 | 0.0005029 | 0.0002525 | 0.0003618 | 0.0006398 | 0.0001658 | 8.896E-05 |
| D-mannose | 0.0002378 | 0.0006316 | 0.0001008 | 0.0187004 | 0.021654 | 0.0219224 | 0.0023734 | 0.0179957 | 0.0282647 | 0.0523408 | 0.0208604 | 0.0132487 |
| glyceric-acid | 0 | 0 | 0 | 8.71E-05 | 6.31E-05 | 0 | 2.64E-05 | 2.44E-05 | 8.82E-05 | 0 | 0 | 0 |
| glycerol | 0.2415517 | 0.2589466 | 0.1757083 | 0.0970846 | 0.107968 | 0.0910312 | 0.0625587 | 0.0894774 | 0.0778123 | 0.3485817 | 0.074501 | 0.1089492 |
| glycine | 0.0002333 | 0.0006119 | 0.0002647 | 0.0031543 | 0.0045326 | 0.0022404 | 0.000678 | 0.0004237 | 0.0004325 | 0.0005113 | 0.0017217 | 0.001313 |
| glycolic-acid | 0.0360998 | 0.0518875 | 0.0330327 | 0.029839 | 0.0288112 | 0.0401123 | 0.0341069 | 0.0369849 | 0.0265976 | 0.0302889 | 0.0322629 | 0.0365439 |
| L-(+)lactic-acid | 0.036191 | 0.0463051 | 0.0297873 | 0.5276036 | 0.2932605 | 0.4026001 | 0.0516654 | 0.0405605 | 0.020525 | 0.0502219 | 0.0527126 | 0.0279373 |
| lactobionic-acid | 0 | 0 | 0 | 0.000909 | 0.0001712 | 0.0024329 | 0 | 0.0335276 | 0 | 0.0082407 | 0.0122138 | 0.0061069 |
| lauric-acid | 8.52E-06 | 1.89E-05 | 1.57E-05 | 0.0005824 | 0.0031288 | 0.000776 | 0.0014244 | 0.0005803 | 0.0002801 | 0.0003974 | 0.000615 | 0.0003615 |
| linoleic-acid | 0 | 0 | 0 | 0.000568 | 0.000813 | 0.0020124 | 0 | 0 | 0.0003335 | 0 | 0 | 0 |
| L-serine | 0 | 0 | 0 | 0.000537 | 0.0026795 | 0.0004851 | 8.04E-05 | 0.0003094 | 0.0002657 | 0.0001084 | 6.99E-05 | 3.494E-05 |
| L-threonine | 0.0002848 | 0.0002812 | 8.66E-05 | 0.0004625 | 0.0011594 | 0.0011908 | 0.0001838 | 0.0001687 | 5.53E-05 | 3.94E-05 | 6.60E-05 | 4.621E-05 |
| methyl-oleate | 3.11E-05 | 1.78E-05 | 1.78E-05 | 0 | 0.0001514 | 0 | 0.0004624 | 0.0011826 | 0.0018001 | 0.0001752 | 0.0004457 | 0.0002229 |
| methyl-palmitoleate | 1.40E-05 | 0 | 0 | 0.0005396 | 0.0001567 | 0.0002687 | 0.0001743 | 0.0003584 | 0.000703 | 0 | 0.0005466 | 0.0002733 |
| methyl-beta-D-galactopyranoside | 0.000708 | 0.0009134 | 0.0014717 | 0.0096653 | 0.0032213 | 0.0029318 | 0.006645 | 0.002252 | 0.0088489 | 0.036544 | 0.0039471 | 0.0630345 |
| myristic-acid | 0.0002785 | 7.23E-05 | 5.31E-05 | 0.0020326 | 0.0069224 | 0.0014281 | 0.0028736 | 0.0004353 | 0.0017387 | 0.0003711 | 0.0001074 | 0.0001458 |

| | | | | | | | | | | | | |
|---|---|---|---|---|---|---|---|---|---|---|---|---|
| oleic-acid | 0 | 0 | 0 | 0.000568 | 0.0014051 | 0.0009043 | 0.0001668 | 8.22E-05 | 9.75E-05 | 5.82E-05 | 5.13E-05 | 3.24E-05 |
| palmitic-acid | 0.0002296 | 0.0001475 | 0.0002188 | 0.0374743 | 0.0910907 | 0.0248237 | 0.1139821 | 0.0674624 | 0.0250011 | 0.0121348 | 0.0475561 | 0.0400747 |
| phosphoric-acid | 0.0003364 | 0.0001884 | 0.004448 | 0.0529177 | 0.1513675 | 0.0022921 | 0.0024536 | 0.0018253 | 0.0031921 | 0.0013221 | 0.0004536 | 0.0006587 |
| phytol | 0.0015121 | 0.0006489 | 0.0008208 | 0.0009258 | 0.0013585 | 0.0033078 | 0.0022071 | 0.0031476 | 0.0026063 | 0.0013431 | 0.001083 | 0.0007628 |
| porphine | 0.166477 | 0.1728083 | 0.1655553 | 0.2821455 | 0.8910706 | 0.3193928 | 0.0359462 | 0.1533144 | 0.1657674 | 0.4394592 | 0.5147948 | 0.3248571 |
| squalene | 0.0007979 | 0.0005157 | 0.0001066 | 0.0027729 | 0.009713 | 0.0091349 | 0.001312 | 0.001884 | 0.0020152 | 0.0022728 | 0.0030083 | 0.0017407 |
| stearic-acid | 0.0008543 | 0.0008467 | 0.0005525 | 0.0963554 | 0.0514845 | 0.0100589 | 0.0786964 | 0.01942 | 0.0505867 | 0.0507149 | 0.0454849 | 0.0288073 |
| Sucrose | 0.0231378 | 0.0175962 | 0.0214683 | 0.009134 | 0.0088664 | 0.0073338 | 0.0117813 | 0.050573 | 0.0372714 | 0.0117805 | 0.0150942 | 0.0116737 |
| tagatose | 8.98E-05 | 0 | 1.27E-05 | 0.0004785 | 0.0004033 | 0.0004886 | 0.0003461 | 0.0002824 | 0.0002947 | 0.001229 | 0.0001107 | 6.597E-05 |
| talose | 0.000708 | 0.0007927 | 0.0005765 | 0.001178 | 0.0020365 | 0.001528 | 0.0018132 | 0.0017388 | 0.0027008 | 0.0073372 | 0.0026761 | 0.0013522 |
| urea | 7.25E-05 | 0.0002139 | 0.0015832 | 0.0046337 | 0.0135274 | 0.0102084 | 0.0094748 | 0.0035561 | 0.0038124 | 0.0054548 | 0.0036546 | 0.0019411 |
